# Leprosy treatment: Can we replace opinions with research?

**DOI:** 10.1371/journal.pntd.0008636

**Published:** 2020-10-08

**Authors:** David M. Scollard

**Affiliations:** Retired, Wilbraham, Massachusetts, United States; Swiss Tropical and Public Health Institute, SWITZERLAND

The current debate in this journal regarding recommendations for leprosy treatment, represented by the comments of highly respected colleagues Lockwood and colleagues [1], and Kumar and colleagues [2], illustrates a longstanding, serious shortcoming in this arena: There is a multitude of expert opinions but a paucity of fundamental research.

Expert opinion has long been at the heart of recommendations for leprosy treatment. The three main drugs in the multidrug treatment (MDT) regimen (dapsone, rifampin, and clofazimine) were selected by an expert committee of the World Health Organization (WHO) in 1981 [3]. There was no randomized clinical trial. Antimicrobials could not be evaluated in culture, since *Mycobacterium leprae* is not cultivable. However, the expert committee did have basic microbiological evidence from the best method available in the 1960s and 1970s: the mouse footpad assay (mfp) (reviewed in [4]). This method is expensive, technically challenging, and requires maintaining dozens or hundreds of mice for many months for even a modest study. It is also very slow; results for “culture and sensitivity” for *M*. *leprae* by mfp studies usually require 12 months or more. Nevertheless, it was the best method available 40 to 50 years ago, and it provided, for the first time, a means to assess the efficacy of anti–*M*. *leprae* agents before conducting a clinical trial.

The MDT regimen has worked quite well, but the repeated revision of the World Health Organization’s recommendations regarding MDT (reviewed in [5]), mostly recommending shorter and shorter duration, is an implicit admission that MDT is not optimal. Several small studies have tried to evaluate alternative regimens [6,7], sometimes using different drug combinations such as rifampin, ofloxacin, and minocycline [8]. But one of the fundamental outcome measures most often used to support each new iteration of MDT is relapse, which is a very poor measure in leprosy.

First, relapse is difficult to substantiate: Slit skin smears or biopsies are required to demonstrate a decline in bacterial load during treatment, followed by an increase after completion of treatment [9]. The skin smear method is more than 80 years old [10] and has many drawbacks. Fluid from a superficial incision in the skin is smeared on a slide and stained for acid-fast bacilli. The number of bacilli is estimated manually and reported as a bacterial index (BI). The BI will decline slowly after initiation of treatment. However, a decline in BI does not directly measure bacterial killing. but, rather, documents the slow removal of dead bacilli from tissue; the carcasses of dead *M*. *leprae* remain in the skin for years after the bacilli have been killed. Another application of skin smears was the estimation of the percentage of *M*. *leprae* having a “solid-staining, beaded or granular” morphology, reported as a morphologic index (MI). This was hypothesized to be a measure of viability of *M*. *leprae*, based on mouse footpad assays of questionable sensitivity for such a conclusion [11]. However, with repeated use and mention in published papers the “MI hypothesis” was accepted as true for many years. Skin smear results can vary markedly due to variations in obtaining the specimen, staining technique, and observer variation, making it very difficult to standardize the BI and MI in studies lasting several years. The BI is used much less frequently today, and efforts to assess the MI were abandoned long ago in most Hansen’s disease (HD) programs. The MI clearly would be an unwieldy and inappropriate method to use to assess the killing of *M*. *leprae* in studies of new MDT regimens today. Notably, WHO no longer recommends skin smears. However, without some form of evidence of changes in bacterial load, there is no objective definition of relapse.

Secondly, relapse in leprosy peaks 10 to 15 years after completion of treatment [12,13] because the multiplication rate of *M*. *leprae* is exceedingly slow. For logistical and financial reasons, studies of such long duration are seldom feasible. Therefore, most studies cited to support shorter MDT regimens lack sufficient follow up to convincingly assess relapse. Hence, many expert physicians are unconvinced by the studies and recommendations, and the debate continues.

Clearly, a newer, better MDT regimen is desirable. Additional agents bactericidal for *M*. *leprae* have been identified in the 40 years since MDT began (reviewed in [5]), and these could now be considered in new MDT regimens. But using relapse as an indicator would require trials involving hundreds of patients for a decade or longer, and the uncertainty about whether treatment had been effective could raise serious ethical concerns. Alternatively, using the mfp assay to assess bacterial killing in biopsies from large clinical trials would be very costly and slow (although faster than waiting for relapses). It is futile, and very probably not fundable, to propose to test new regimens using obsolete tools developed 40 to 80 years ago.

Recent technological advances may offer a better path forward. A molecular viability assay has been developed that measures levels of mRNA from *M*. *leprae*, enabling assessment of the killing efficacy of drug regimens in leprosy [4,14]. Using this assay to assess anti–*M*. *leprae* agents in the mouse model, for example, demonstrated that the most effective agent is rifabutin, followed by other agents that are in current use [14]. With this assay, the viability of *M*. *leprae* after a selected treatment regimen can be assessed directly from patients’ skin biopsies in a shorter time and at a lower cost than the mfp assay [15]. This molecular method could be used to screen different proposed MDT regimens in well-designed studies in small patient cohorts, to identify the best candidate drug regimens. With this method the required duration of treatment can also be determined empirically: There is nothing magic about 6, 12, or 24 month MDT protocols. The best candidate regimen(s) from such screening could then be evaluated in a more robust manner in larger trials.

This is an opportune time for WHO, Global Partnership for Zero Leprosy, and other organizations that provide global leadership in leprosy to promote and support investigators to propose different candidate MDT protocols and, then, use this assay to screen them in small clinical trials to determine which is the most promising. Such an approach would provide new empirical data upon which to base MDT recommendations for leprosy for the 21st century. Scientific evidence could finally replace expert opinion as the basis for recommending optimal treatment of infection with *M*. *leprae*.
